# Design and Implementation of an Electronic Front-End Based on Square Wave Excitation for Ultrasonic Torsional Guided Wave Viscosity Sensor

**DOI:** 10.3390/s16101681

**Published:** 2016-10-12

**Authors:** Amir Rabani

**Affiliations:** Advanced Manufacturing Technology Research Group, Faculty of Engineering, University of Nottingham, University Park, Nottingham NG7 2RD, UK; Amir.Rabani@nottingham.ac.uk; Tel.: +44-115-951-4079

**Keywords:** torsional guided waves, viscosity sensor, ultrasonic front-end, square wave excitation

## Abstract

The market for process instruments generally requires low cost devices that are robust, small in size, portable, and usable in-plant. Ultrasonic torsional guided wave sensors have received much attention by researchers for measurement of viscosity and/or density of fluids in recent years. The supporting electronic systems for these sensors providing many different settings of sine-wave signals are bulky and expensive. In contrast, a system based on bursts of square waves instead of sine waves would have a considerable advantage in that respect and could be built using simple integrated circuits at a cost that is orders of magnitude lower than for a windowed sine wave device. This paper explores the possibility of using square wave bursts as the driving signal source for the ultrasonic torsional guided wave viscosity sensor. A simple design of a compact and fully automatic analogue square wave front-end for the sensor is also proposed. The successful operation of the system is demonstrated by using the sensor for measuring the viscosity in a representative fluid. This work provides the basis for design and manufacture of low cost compact standalone ultrasonic guided wave sensors and enlightens the possibility of using coded excitation techniques utilising square wave sequences in such applications.

## 1. Introduction

There has been an increasing demand for real-time monitoring and control of fluids both in process and in storage over the past 20 years. Sensor systems for real-time and in situ applications, which are capable of withstanding the process environment coupled with meeting health and safety requirements, are increasingly required by many industries. Many fluids found in industry are complex in nature. They can contain suspended solid or liquid particles, and more generally can exhibit complex rheological behaviour including time-, shear-, temperature-, and pressure dependence. These complex properties highlight the need for robust and innovative sensor system design for process industries. Exposure to hostile and hazardous process conditions, such as plant vibrations, fouling, cleaning agents, and toxic compounds pose additional challenges in instrument design.

Among the rheological properties of fluids, viscosity is an integral and necessary component of many quality control procedures in the processing of complex liquids [1]. Monitoring viscosity on-line and in situ provides real-time data which can be used to optimise the process and support product quality. Finding and developing a method and instrument for in situ measurements of viscosity for different environments has been of continuing concern over many years in the area of industrial process control. Several technologies are commercially available for viscosity measurements on-line and in situ but have their own constraints [2,3,4]. They are normally expensive and need frequent and costly maintenance. To contain costs and provide disposability, the choice of sensor system and the design of the supporting electronic platform are important. 

Recently, much attention has been paid to ultrasonic methods, particularly for measuring properties of different materials. Many instruments and techniques have been developed on measuring rheological properties of fluids using ultrasound during past decades to find the density and viscosity of fluids [5,6]. In particular, ultrasonic guided wave sensors have been used to measure viscosity and/or density in liquids by many researchers [7,8,9,10,11,12,13,14,15,16,17,18,19,20,21]. They can be formed into robust instruments which are inexpensive enough to be disposable when applied to hazardous materials, or can be used on-line in a process. They have advantages over conventional systems which are delicate, expensive, and not suitable for operation in a process line.

The most popular ultrasonic guided wave technique for measuring fluids’ viscosity and density is based on the application of ultrasonic torsional wave propagation along a cylindrical rod or a thin wire waveguide, which is immersed in a viscous fluid. In operation, a torsional stress wave propagates along a solid or hollow waveguide while it is submerged in a fluid. The boundary layer of the fluid is alternately accelerated and decelerated as the torsional wave travels along the waveguide and reflects back from its distal end. The echoes thus received are amplified and processed to extract the propagation velocity and/or the attenuation of the waves in the waveguide. Both velocity and attenuation are affected by coupling between the motions of the waveguide surface associated with the propagating wave in the rod and motions in the liquid in contact with the waveguide. Under the common condition that phase speed within the rod is greater than the speed of viscous waves in the surrounding fluid, such coupling will cause radiation of energy into the fluid, reducing both speed and amplitude of the guided wave.

McSkimin [7] made the first application based on this property of travelling torsional waves to measure fluid dynamic shear viscosity and stiffness of viscous liquids. Later, Lynnworth [22] designed a sensor based on the velocity of the propagating torsional stress waves to measure fluid density. A quantitative theory was developed by Bau [23] and optimised by Kim and Bau [24] and Fan et al. [25]. Kim and Bau [9] and Shepard et al. [14] applied this theory further using rectangular and circular cross-sections to measure density and viscosity simultaneously. Their work led to successful viscosity measurements of simple fluids. Costley et al. [12] and Vogt et al. [15] developed methods for measuring viscosity based on the attenuation of the fundamental torsional mode for measuring the viscosity of fluids. Their measurements showed good agreement with viscosity measurements using conventional rheometers when testing general purpose viscosity standards. Kim et al. [10] derived an asymptotic formula for attenuation of the fundamental torsional mode in a rod loaded by viscous fluid. Based on their model Rabani et al. [18] proved that the attenuation of the fundamental torsional wave is more sensitive to the viscosity of a surrounding fluid compared to the velocity of the guided fundamental torsional wave and derived an explicit expression to calculate viscosity in a fluid of known density from the measured attenuation. They showed successful viscosity measurements of Newtonian fluids; however, anomalous behaviour was observed in the measurement of the viscosity of engine, gear, and silicone oils. This anomalous behavior was explained by molecular relaxation phenomena, which has a significant effect on the viscosity of polymer-based fluids with long chain molecules. They extended their work further by developing an analytical model to estimate the torsional guided wave viscosity sensor shear rate [26].

It would thus appear that the ultrasonic torsional guided wave technique can be successfully employed to measure the viscosity of wide range of fluids. The electronic systems used in the works outlined above are mainly designed to support a wide range of guided wave experiments in the laboratories and the experiments were mainly done using a windowed sine wave burst as the excitation signal. These systems are bulky and expensive, and they can provide for many different signal settings which are not required for monitoring viscosity online and in situ in process plants. However, a self-contained sensor system based on bursts of square waves instead of sine waves would have a considerable advantage in terms of manufacturing simplicity and cost. This paper explores the possibility of using square wave bursts as the driving signal source and proposes a simple design of a compact and fully automatic analogue square wave front-end for the ultrasonic torsional guided wave sensor.

## 2. Sensor Excitation Method

The ultrasonic torsional guided wave viscosity sensor consists of a waveguide and a torsional wave transduction system [26]. The waveguide is an elastic cylindrical rod which is in a test fluid. The torsional waves are excited by utilizing ultrasonic transducers. An ultrasonic torsional wave transducer converts electrical energy into acoustic energy in transmission mode when its active element is excited by a voltage or current signal; on the other hand, in reception mode the acoustic energy of the returning torsional ultrasonic wave is converted into an electrical signal. 

Piezoelectric materials, particularly piezoelectric ceramics, are extensively in use for generating and receiving ultrasonic waves in medical imaging, non-destructive testing, and process control applications [27]. These materials are also highly durable; they have high tensile strength and their polarization geometry can be designed-in. These features make them more suitable for sensor design. PIC 255 piezoelectric ceramic plates (PI Ceramic GmbH, Lederhose, Germany) which operate in thickness shear mode are well suited for use in the ultrasonic guided wave torsional sensor transducer to generate torsional modes. PIC255 is a modified PZT with 350 °C Curie temperature, 2400 permittivity in the polarization direction, 0.66 coupling factor for the thickness shear oscillation and 550 × 10^−12^ N/C shear charge coefficient. It is suitable for low-power ultrasonic transducers due to its low mechanical quality factor (i.e., 80), which means larger power for off-resonant excitations [28], and low temperature coefficient (i.e., 0.004/K). 

**Figure 1:**
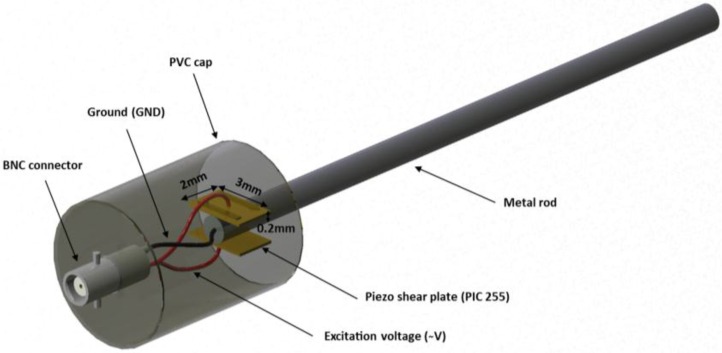
Ultrasonic torsional guided wave viscosity sensor.

In order to excite torsional guided waves in the sensor rod, the design based on the shear to torsional mode conversion was adapted [29]. Hence, two rectangular shear plates (3 mm × 2 mm × 0.2 mm) are coupled on different sides at one end of the axially-elongated rod with direction of polarization opposed each to other (Figure 1); to provide the required robustness, the whole assembly is encapsulated within a PVC cap using general purpose epoxy resin. 

**Figure 2:**
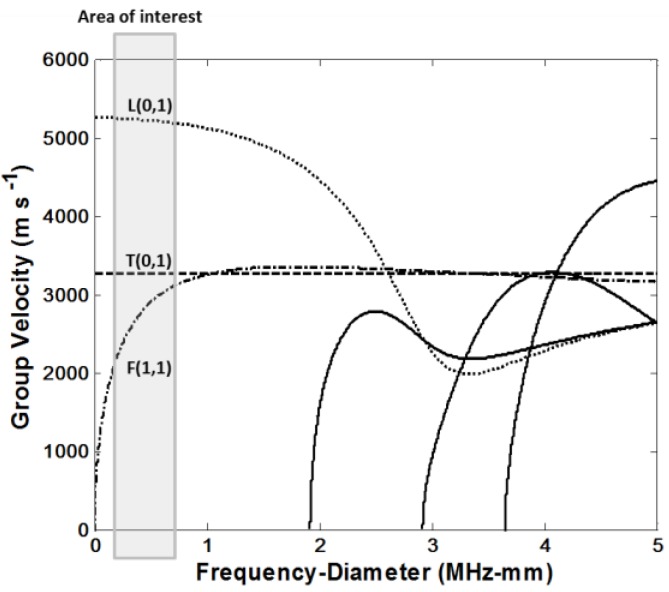
Dispersion curves for cylindrical carbon steel rods: Group velocity versus frequency-diameter product.

Once the voltages applied to the plates in a direction perpendicular to the direction of the polarization, shear stresses couple in a plane perpendicular to the axis of the rod which generates a torsional wave in the rod. For large diameter rods, it is possible to use more than two shear plates all placed perpendicular to the axis and around the circumference of the rod such that the adjacent plates have the same polarization and vibrate in the circumferential direction. In principle, shearing piezoelectric transducers, if themselves perfect, and if perfectly attached to the sensor rod, should excite only a torsional mode. However, such perfection is difficult to achieve in practice and some energy will exist in non-torsional modes such as L(0,1) and F(1,1) in Figure 2, for example. It is therefore necessary that the frequency-diameter product be such that the required torsional mode travels at a speed that is significantly different from that of the unwanted modes—in order to allow time gating of the torsional signal for further processing. Referring to Figure 2, this would require an operating frequency well below 1 MHz for the sensor with 1mm diameter steel rod where T(0,1) mode has higher speed compare to F(1,1) and lower speed compare to L(0,1) modes. The maximum centre frequency of the induced torsional wave in the torsional viscosity sensor with 1mm diameter steel rod and two rectangular piezoelectric shear transducers with 3 mm length, 2 mm width, and 0.2 mm thickness is lying approximately in the 500 to 700 kHz range which perfectly satisfies operability requirement for the torsional viscosity sensor. 

The frequency response of the tone-burst excitation signal is narrow band with suppressed side lobes, so it is perfectly matched to the requirements for torsional guided wave applications where the excitation of fundamental torsional mode is of interest. Square wave burst signals have several harmonics and stronger side lobes. However, only the first harmonic is within the frequency response of the torsional sensor transducers and thus higher harmonics are not likely to have any effect on the excited modes in the sensor. Therefore, it is possible to use the first harmonic spectrum in the attenuation measurements, since this has a similar frequency spectrum to the tone-burst signal. 

**Figure 3:**
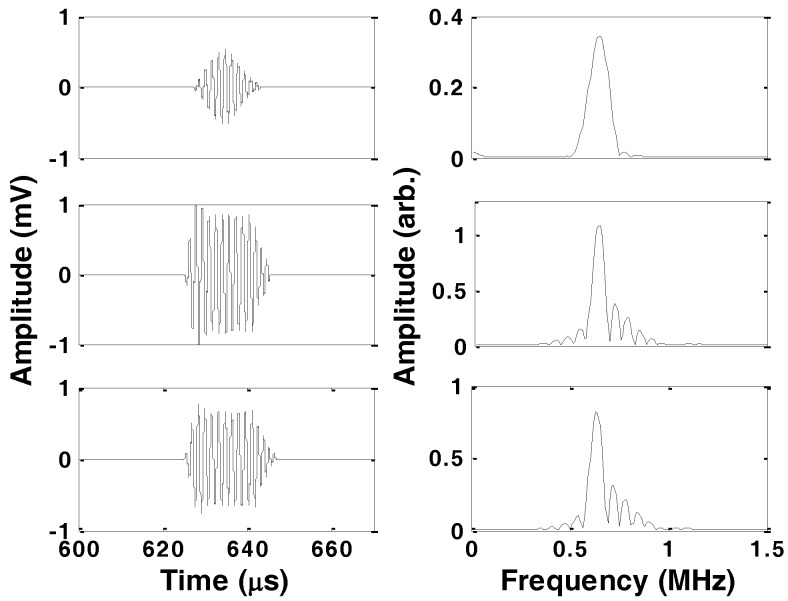
Time domain (**left**) and amplitude spectrum (**right**) of the received T(0,1) mode, from 10 cycles of tone-burst (**top**), unipolar square wave burst (**middle**), and bipolar square wave burst (**bottom**) excitation signals in 1 mm diameter carbon steel rod at frequency of 625 kHz after travelling down the immersed sensor rod in a viscous liquid and reflection from the distal end.

Figure 3 shows the time and frequency plots of the received signals for tone-burst, unipolar, and bipolar square wave 10-cycle burst excitation signals in 1 mm carbon steel rod at 625 kHz immersed in a viscous liquid. The received signal is attenuated and deformed due to losing energy by travelling through the immersed sensor rod and capacitive nature of the transducers, respectively. In the frequency domain, the higher harmonics that are expected from the square wave bursts are greatly suppressed. The lobes which are clearly observable around the first harmonic for unipolar and bipolar square wave bursts result from the fact that the window length for the transmitted burst was 17.5 μs; the reciprocal of this implies a frequency domain lobe width of 57 kHz.

**Figure 4:**
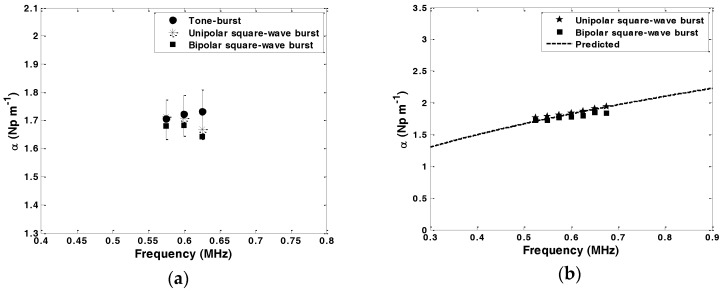
Attenuation measurements for the T(0,1) mode for 1mm diameter carbon steel rod immersed in olive oil (**a**) using tone-burst, unipolar square wave burst, and bipolar square wave burst as excitation signals. Average values of five recordings are shown as symbols. Solid error bars represent the standard deviation of the measurements using the tone-burst; (**b**) using unipolar and bipolar square wave bursts as excitation along with predicted values.

To evaluate the sensor operation with different excitation signals, the probe was immersed in a Newtonian sample liquid with consistent behavior across a range of shear rates and frequencies (i.e., olive oil), and the three waveforms (toneburst, unipolar, and bipolar square wave bursts) each 10 cycles long were excited using an arbitrary function generator (DUI, NDT Solutions Ltd, Chesterfield, UK). Five attenuation measurements at each of three different frequencies (575 kHz, 600 kHz, and 625 kHz) were made using each waveform in random order. In order to measure the guided wave attenuation in the sensor rod at the given frequencies, the sensor was immersed in the test liquid and the echo signals from the distal end of the sensor rod were recorded. The received signals from the sensor were amplified up to 57 dB, digitised at 50 MHz, and averaged 100 times by the same arbitrary function generator. The received time domain signals were transformed into the frequency domain by FFT. The attenuation coefficient is calculated from the frequency domain relationship:

(1) $$\alpha \left(\omega \right)=\frac{1}{2l}\ln\frac{X_{\mathrm{CAL}}\left(\omega \right)}{X\left(\omega \right)}$$

where, *l* is the sensor length, and *X*_CAL_(*ω*) and *X*(*ω*) are the frequency domain amplitudes of the received signals from a sensor in air/vacuo and the received signal from an immersed sensor in the test liquid, respectively. The received signals from an immersed sensor in a liquid diminish in amplitude compared to the signals received from an un-immersed sensor. The sensor length was set so as to bring the measured loss as close as possible to 1 Np to achieve the optimum condition for minimum error in attenuation [18]. The average value of the measured attenuation on each waveform at each frequency is calculated and shown in Figure 4a. At each frequency, the standard deviation of the attenuation for each group of five readings was calculated for each signal type. These were found to be less or equal to 6.7%, 4.4%, and 8.5% for tone-burst, unipolar square wave burst and bipolar square wave burst signals, respectively. This is shown on Figure 4a for the tone-burst as solid error bars. 

The tone-burst signal has gained general acceptance as the appropriate driving signal for guided wave applications. As mentioned before, it is a narrowband signal without side lobes. In attenuation measurement, a single frequency from the frequency domain signal is used, in which case the shape of the signal does not have an effect on the measurement. Hence, as can be seen in Figure 4a, the differences between attenuation determined using different driving signals are statistically insignificant. The only difference between unipolar and bipolar square wave signals is the DC component associated with the unipolar square wave signal. Since the piezoelectric shear plates used in the torsional probe are capacitive and act as bandpass filters, the unipolar square wave shows the same behaviour as the bipolar square wave. Comparing the attenuation measured by unipolar and bipolar square wave burst signals shows that either of the signals can replace the tone-burst signal.

To further validate the applicability of the unipolar and bipolar square wave burst excitation signals in the operation of the ultrasonic torsional guided wave viscosity sensor, attenuation measurements for the T(0,1) mode in 1mm diameter carbon steel rod immersed in olive oil at frequencies ranging from 525 kHz to 675 kHz with intervals of 25 kHz were carried out. As illustrated in Figure 4b, there is a good agreement between the measured and the predicted attenuation values calculated from the attenuation expression for T(0,1) derived by Kim and Bau [9]. Having established that no significant difference exists in the attenuation when using different driving signals, and that the measured attenuation from different signals agrees with expectation, the unipolar square wave is the best candidate on the grounds of cost and simplicity of design.

## 3. Electronic Front-End System Design

**Figure 5:**
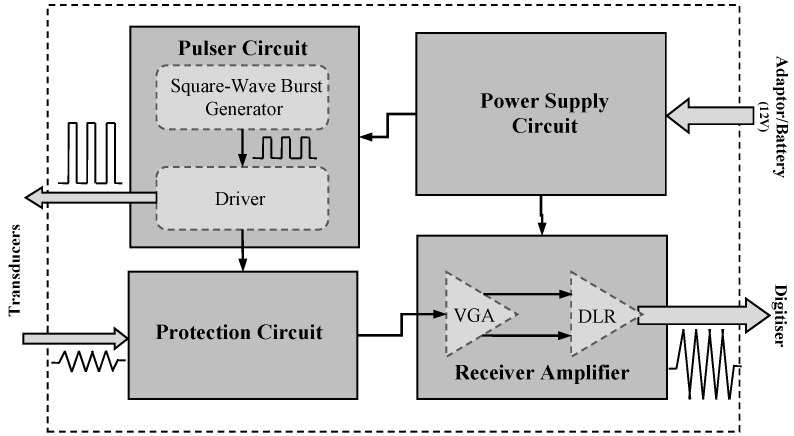
Schematic of the ultrasonic unipolar square wave front-end.

There are trade-offs associated with designing ultrasound front-end circuits which relate to performance requirements, underlying physics, and cost. It is essential to understand the specifications that are of particular importance and their potential effects on system performance that may limit design choices. In the following, a simple implementation of a square wave ultrasonic front-end for use with the ultrasonic torsional guided wave viscosity sensor is described. The proposed circuit design contains four major components: a pulser circuit, a protection circuit, a receiver amplifier, and a power supply. These analogue electronic components are key to the overall system performance. Their characteristics define the limits to that performance in terms of unwanted noise and signal distortions. Figure 5 shows a schematic of the proposed front-end system. 

The pulser circuit acts as a transmitter which generates the basic square wave burst signal in two steps: first a unipolar square wave is generated and this is then amplified by a high voltage driver that connects to the transducers. On the receiver side, there is a protection circuit which blocks the high voltage signals from the transmitter. It is followed by a low-noise variable gain amplifier and a differential line receiver. The amplified signal can then be sent to a digitiser for further processing. The power supply circuit provides the different voltages required for all the modules in the system. In the proposed system there is a capability to control the number of cycles, frequency, and repetition rate of the excitation signal, and also the gain of the receiver amplifier. These provide the ability to generate unipolar square wave bursts with different characteristics for experimental purposes. However, in real-time and in situ applications the ultrasonic torsional guided wave viscosity sensor will only require excitation signals with fixed frequency and number of cycles. A front-end system with fixed frequency and number of cycles provides possibility for a compact design with no pre-settings that can be easily integrated into the sensor package for standalone permanent/remote installations.

### 3.1. The Pulser Circuit

**Figure 6:**
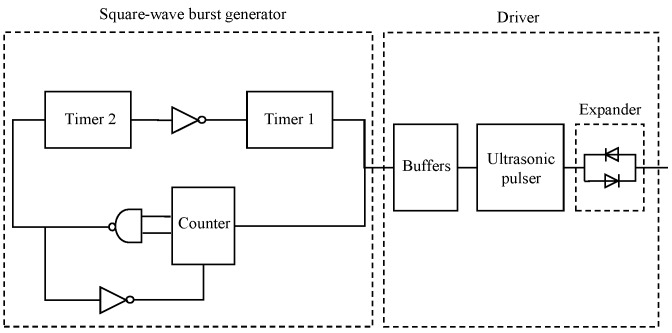
Simplified diagram of the pulser circuit.

The primary objective of the pulser circuit is to generate and amplify a burst of square wave signals for transmission. This is achieved by a two-stage design, namely a square wave generator module and the driver module (Figure 6). In the first stage, the square wave burst generator initiates a sequence of unipolar pulses (square wave bursts) with maximum amplitude of 5 V. In the next stage, the driver amplifies the sequence of pulses to 72 V amplitude. 

The square wave burst generator is formed of two CMOS timers (e.g., LMC555, National Semiconductor Corp., Santa Clara, CA, USA), a binary counter (e.g., HFE 4520 B, NXP Semiconductors Netherlands B.V., Eindhoven, The Netherlands), and three logic gates (Figure 6). Timer 1 and Timer 2 are configured as astable and monostable multivibrators, respectively. Timer 1 generates the square wave burst at frequencies which can be set up to 3 MHz. Timer 2 controls the repetition rate of the burst. The counter and the logic gates set the number of cycles for each burst. The frequency and repetition rate are settable by changing the timing resistors for Timer 1 and Timer 2, respectively. This has been done using jumper switches on the prototype system in this study for experimental purposes. As mentioned earlier, in industrial applications there is no need for variable frequency and repetition rate and a single timing resistor for each timer will be sufficient.

An external 5 V trigger signal starts the driving process; Timer 1 starts working as a free running multivibrator, generating the square wave signals. The counter counts the number of pulses generated by Timer 1 and it triggers Timer 2 as soon as it reaches the maximum number of pulses set by the logic gates. Timer 2 generates a single output pulse which resets Timer 1. The width of the output pulse from Timer 2 defines the repetition rate of the square wave burst. The circuit generates a continuous sequence of square wave burst packets automatically.

The driver consists of two input buffers, an ultrasonic pulser and an expander circuit. The signals are fed to the ultrasonic pulser through inverting and non-inverting buffers. The ultrasonic pulser amplifies the square wave signals to an appropriate level to drive the transducers through the expander circuit. In common with many ultrasonic driver circuits, the expander circuit consists of two parallel diodes in opposite directions. It conducts high-voltage amplified signals generated by the pulser and connects the pulser to the transducers during the transmission phase. Conversely, it stops conduction at the time of termination of the burst sequence and isolates the pulser from the transducer during the reception phase. In this work, the Supertex HV748 (Supertex Inc., Sunnyvale, CA, USA) is used as the ultrasonic pulser. It is a monolithic high speed, high voltage, ultrasonic transmitter pulser. It is designed as a high voltage driver for piezoelectric transducers and other applications. Its circuit consists of controller logic circuits, a level translator, gate driving buffers, and a high current and high voltage MOSFET output stage. The output stages of this integrated circuit can provide output voltages up to ±75 V and output currents up to ±1.8 A. It can operate at frequencies up to 20 MHz depending on the load capacitance.

In the ultrasonic torsional guided wave viscosity sensor applications, it is required to generate high amplitude ultrasonic signals to have a higher signal to noise ratio, particularly for viscosity measurements of highly viscous liquids for which the attenuation of the signal is large. Hence, the ultrasonic pulser is provided with a maximum 72 V voltage at its high voltage power supply pin. This enables the generation of unipolar sequences with a maximum 72 V amplitude at the output. 

**Figure 7:**
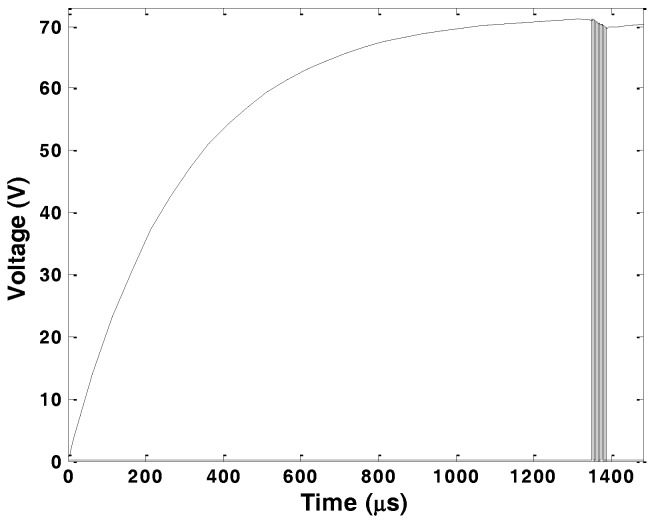
Decoupling capacitor charging time and change in the voltage at pulser high voltage power supply pin (**dashed-dot line**). 10 cycle output (**solid line**).

Under the sequence of pulses, the HV748 internal MOSFET-gate-drives switch several times and this draws large instantaneous currents and results in a voltage drop due to the impedance of the voltage source and the connection between the voltage source and the ultrasonic pulser. Thus, the decoupling capacitor should be chosen carefully to maintain the supply voltage level at the ultrasonic pulser. This capacitor introduces a minimum delay to the system for generating pulses with the maximum amplitude due to initial charging to the maximum voltage. A 168 nF decoupling capacitor was used in the proposed design. Figure 7 shows the minimum charging time required to reach to 99% of the maximum voltage of 72 V. The reduction in the pulse amplitude after 10 cycles of the square wave pulses is less than 1.5%.

In the measurement of torsional wave attenuation, all the measurements are made using a reference signal and so the reduction in the pulse amplitude is present in both reference and measurement signals and does not affect the measurement result.

### 3.2. The Protection Circuit

**Figure 8:**
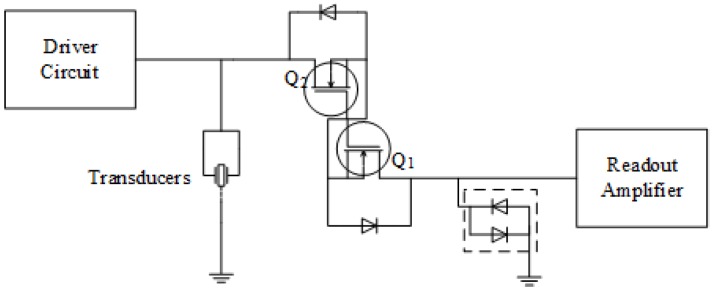
Schematic of the automatic protection circuit.

The driver circuit generates high-voltage square wave burst packets (maximum 72 V) on the node where the transducers are connected. The receiver amplifier circuit is designed for relatively low-voltage signals. Hence, to prevent high-voltage square wave bursts from damaging the receiver amplifier, the protection circuit is placed between the transducers and the receiver amplifier circuit. The protection circuit behaves as an open circuit while the system is in the transmission state and as a short circuit while the system is in the reception state. The protection circuit in this design is based on the design by Camacho and Fritsch [30]. The design is fully automatic and has many advantages such as operating without any control signals or bias voltages, low harmonic distortion, low insertion losses, low noise, low power dissipation, high bandwidth, and small size. Figure 8 shows the protection circuit arrangement. It is based on a pair of N-channel depletion MOSFETs.

In operation when the gate-source voltage is very close to zero and the drain-source voltage is less than the difference between the gate-source voltage and the threshold voltage, both MOSFETs conduct with low drain-source impedance. In the presence of a positive input voltage, the diode associated with Q1 conducts, Q2 operates in the saturation region, and the limiter diodes conduct the drain saturation current safely to ground. This circuit also operates with negative input, in which case, Q1 and Q2 swap their roles.

### 3.3. The Receiver Amplifier

A simple two-stage receiver amplifier is designed for low-noise operation, high gain and single-ended output (Figure 5). It is compact and inexpensive, comprising of just two integrated circuits. It consists of a variable gain amplifier (e.g., AD8331, Analog Devices Inc., Norwood, MI, USA) and a differential line receiver (e.g., MAX4444, Maxim Integrated Products, Sunnyvale, CA, USA). The variable gain amplifier has a gain which is settable up to 55.5 dB. It has an embedded preamplifier and symmetrical differential output. It is usable as an ultralow-noise element up to 120 MHz. The differential line receiver has symmetrical differential inputs and a single-ended output and closed-loop gain of +6 dB. When the system is in the reception mode, the signals which are received by the receiver amplifier from the transducers through the protection circuit will be amplified up to 55.5 dB by the variable gain amplifier in the first stage. In the second stage, the differential line receiver converts the differential output from the variable gain amplifier to a single-ended signal for onward transmission via coaxial cable to a distant processing unit.

### 3.4. The Power Supply Circuit

**Figure 9:**
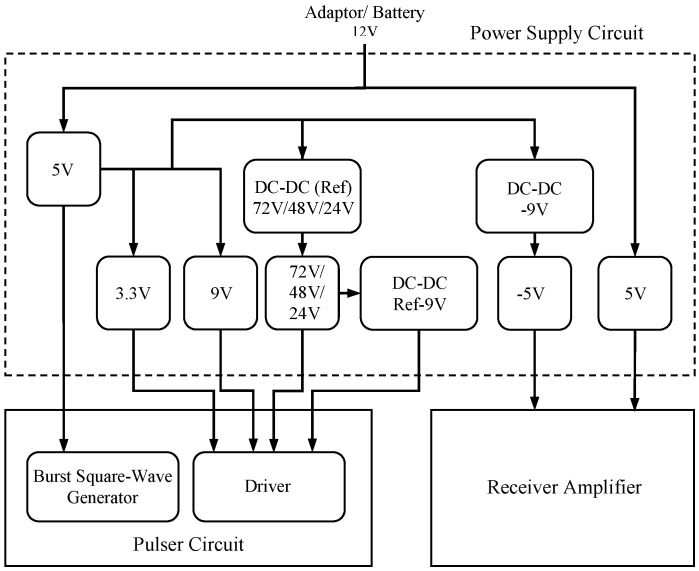
Block diagram of the power supply circuit.

The power supply circuit supplies electrical energy to each electronic module in the system. It is designed to be robust, compact, and inexpensive. It consists of fixed linear regulators, DC-DC voltage convertors, and a high-voltage power supply. It works with 12 V DC external voltage source which can be either an adaptor or a battery. The block diagram of the power supply circuit is given in Figure 9.

A 5 V fixed linear regulator is employed to supply the square wave burst generator module. Two separate fixed linear regulators are provided to supply the pulser with 3.3 V and 9 V voltages. The driver is also supplied with a high-voltage power supply which is formed by a triple output DC-DC voltage convertor (e.g., NMT0572SC, Murata Power Solutions Inc., Mansfield, MA, USA) with voltages of 24 V, 48 V, and 72 V, selectable using jumpers, followed by a high voltage adjustable regulator to regulate the output. Also a DC-DC voltage convertor (e.g., IL0509S, XP Power, Sunnyvale, CA, USA) is used to supply a floating 9 V, a reference to the high-voltage power supply output, which is also required by the driver. The receiver amplifier circuit is supplied with ±5 V fixed linear regulators. The introduction of noise by use of a common power supply for the receiver amplifier and the rest of the system has been avoided by using a separate 5 V linear regulator for the receiver amplifier. 

## 4. System Functional Verification and Analysis 

**Figure 10:**
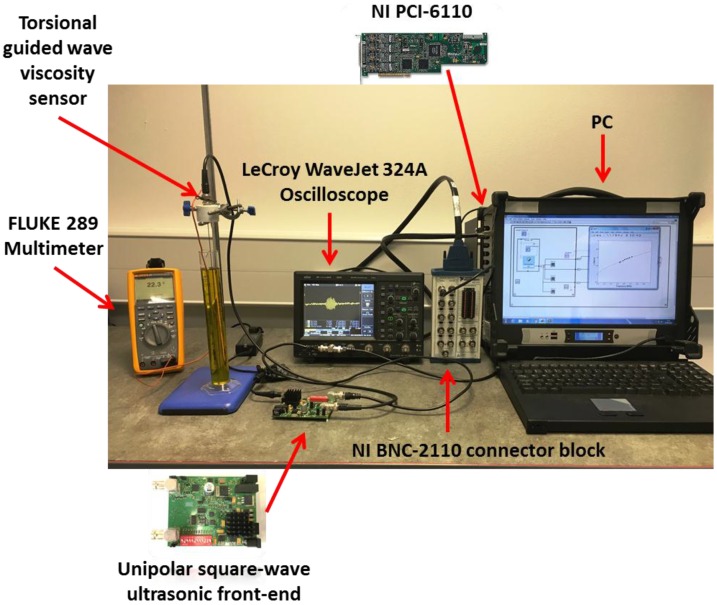
Unipolar ultrasonic square wave front-end experimental setup for torsional guided wave viscosity sensor.

To experimentally verify the performance of the proposed unipolar square wave ultrasonic front-end, the torsional guided wave viscosity sensor was immersed in a test liquid (i.e., extra virgin olive oil) and connected to the designed analogue front-end through a 50 Ω coaxial cable in a temperature controlled room. The amplified analogue received signals from the transducers that were transferred to a portable oscilloscope (WaveJet 324A, Teledyne LeCroy Inc., Chestnut Ridge, NY, USA) for visualization and then to a BNC connector block (NI BNC-2110, National Instruments Corporation, Austin, TX, USA) using a 50 Ω coaxial cables. The BNC connector block was connected to a multifunction data acquisition board (NI PCI-6110, National Instruments Corporation, Austin, TX, USA) for digitization through a shielded cable (NI SH68-68-EPM, National Instruments Corporation, Austin, TX, USA). A digital multimeter (FLUKE 289, Fluke Corporation, Everett, WA, USA) was used to monitor the temperature of the olive oil at the time of the measurements. The received analogue signals were digitised at 5 MHz and averaged 50 times and sent to a PC by a GPIB interface (IEEE 488) for further processing. The experimental setup is illustrated in Figure 10.

**Figure 11:**
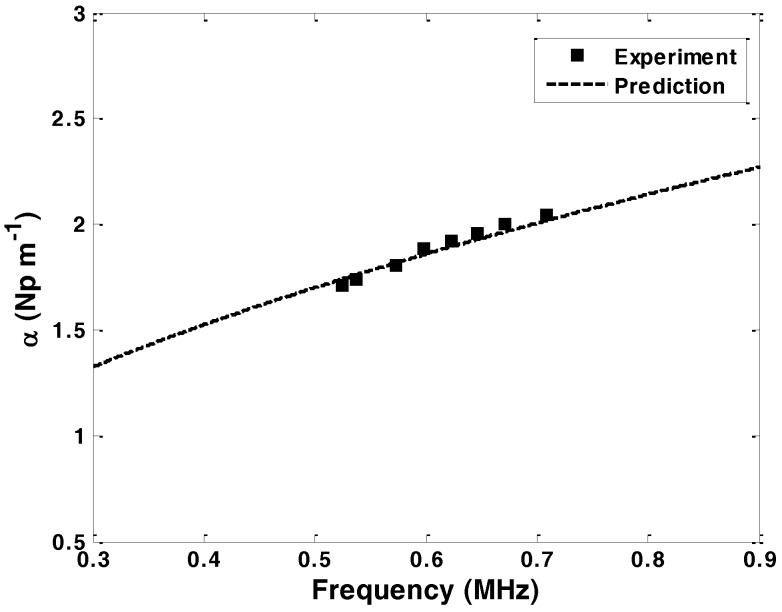
Measured attenuation in 1mm diameter carbon steel rod embedded in an olive oil versus frequency, superimposed on the predicted attenuation curve.

Attenuation measurements were performed at eight frequencies in the range 524 to 708 kHz at 22.3 °C. The attenuation coefficients are calculated using Equation (1). The variation in the attenuation measurements, calculated as a standard deviation over five recordings, was less than ±1.5%. The measured attenuation of 1mm diameter carbon steel rod immersed in olive oil is shown in Figure 11. There is a good agreement between the measured attenuation and the predictions.

Viscosities were calculated from the attenuation readings (eight readings in total) using the explicit viscosity expression given by Rabani et al. [18]. The standard deviations of each group of eight calculated viscosities were less than or equal to 0.6%. Reference viscosity measurements were made for comparison using a Physica MCR 301 (Anton Paar GmbH, Graz, Austria) conventional rotating element rheometer operating in steady shear mode using cone and plate geometry (spindle: CP-50). A total number of 31 measurements were made on the test liquid at shear rates ranging from 0 to 1000 s^−1^. The average viscosity of the torsional guided wave sensor had an error less than 2% compared to the average viscosity measured using the conventional rheometer (Table 1).

## 5. Conclusions

It has been shown that a square wave, instead of a sine wave, burst can be employed in the torsional guided wave viscosity sensor. The electronics for square wave burst signals are less expensive and simpler to implement than those for sine wave signals. However, the square wave burst method is not in itself any better that the tone-burst case. Two types of square waves, namely unipolar and bipolar, were considered for the design. The unipolar square wave was chosen as it requires a simpler electronic platform with fewer components thus reducing the size and the cost of the system.

The design and implementation of an analogue front-end for the torsional guided wave viscosity sensor was presented. The design is simple, fully automatic, and robust. It works without the need of any external controlling signal. The cost of the device is considerably less than that of a conventional ultrasonic wave transmitter and receiver commonly used in many laboratories. It is compact and could be designed to fit into the same package as the transducer head for the torsional guided wave viscosity sensor. The proposed design is flexible for further design improvements; the output gain and the characteristics of the excited wave—such as the burst frequency, number of cycles, and repetition rate—are settable. It can be expanded to work with standalone processing chips such as microcontrollers and FPGAs.

The characteristics and the performance of the designed ultrasonic front-end were verified experimentally. The operation of the system was stable and reproducible within an acceptable error range. The attenuation and viscosity measured by the torsional guided wave viscosity sensor using the designed device was in good agreement with the predicted values and with those measured using a commercial rheometer.

## Figures and Tables

**Table 1:** Average viscosity of the test liquid (torsional sensor vs. conventional rheometer).

Testing Liquid	Density (kg·m^−3^)	Average Viscosity (Pa·s) Physica MCR 301	Average Viscosity (Pa·s) Torsional Sensor
Extra virgin olive oil ^1^	913.0	0.0756	0.0771

^1^ Sainsbury’s Supermarkets Ltd, London, UK.
